# Autonomous robotic ultrasound scanning system: a key to enhancing image analysis reproducibility and observer consistency in ultrasound imaging

**DOI:** 10.3389/frobt.2025.1527686

**Published:** 2025-02-05

**Authors:** Xin-Xin Lin, Ming-De Li, Si-Min Ruan, Wei-Ping Ke, Hao-Ruo Zhang, Hui Huang, Shao-Hong Wu, Mei-Qing Cheng, Wen-Juan Tong, Hang-Tong Hu, Dan-Ni He, Rui-Fang Lu, Ya-Dan Lin, Ming Kuang, Ming-De Lu, Li-Da Chen, Qing-Hua Huang, Wei Wang

**Affiliations:** ^1^ Department of Medical Ultrasonics, Institute of Diagnostic and Interventional Ultrasound, Ultrasomics Artificial Intelligence X-Lab, The First Affiliated Hospital of Sun Yat-Sen University, Guangzhou, China; ^2^ College of Electronic Information, Guangxi Minzu University, Nanning, China; ^3^ Department of Medical Ultrasonics, The Seventh Affiliated Hospital of Sun Yat-Sen University, Shenzhen, China; ^4^ Department of Medical Ultrasound, The First Affiliated Hospital of Guangxi Medical University, Nanning, China; ^5^ Department of Hepatobiliary Surgery, The First Affiliated Hospital of Sun Yat-Sen University, Guangzhou, China; ^6^ School of Artificial Intelligence, Optics and Electronics (iOPEN), Northwestern Polytechnical University, Xi’an, Shaanxi, China

**Keywords:** autonomous robots, ultrasound, reproducibility, consistency, AI

## Abstract

**Purpose:**

This study aims to develop an autonomous robotic ultrasound scanning system (auto-RUSS) pipeline, comparing its reproducibility and observer consistency in image analysis with physicians of varying levels of expertise.

**Design/methodology/approach:**

An auto-RUSS was engineered using a 7-degree-of-freedom robotic arm, with real-time regulation based on force control and ultrasound visual servoing. Two phantoms were employed for the human-machine comparative experiment, involving three groups: auto-RUSS, non-expert (4 junior physicians), and expert (4 senior physicians). This setup enabled comprehensive assessment of reproducibility in contact force, image acquisition, image measurement and AI-assisted classification. Radiological feature variability was measured using the coefficient of variation (COV), while performance and reproducibility assessments utilized mean and standard deviation (SD).

**Findings:**

The auto-RUSS had the potential to reduce operator-dependent variability in ultrasound examinations, offering enhanced repeatability and consistency across multiple dimensions including probe contact force, images acquisition, image measurement, and diagnostic model performance.

**Originality/value:**

In this paper, an autonomous robotic ultrasound scanning system (auto-RUSS) pipeline was proposed. Through comprehensive human-machine comparison experiments, the auto-RUSS was shown to effectively improve the reproducibility of ultrasound images and minimize human-induced variability.

## 1 Introduction

Ultrasound (US) imaging technology holds a prominent position as the most widely utilized imaging modality for clinical intervention and diagnosis, and plays a crucial role in screening and monitoring diseases (Mann et al., 2020; Tamaki et al., 2022). In comparison to computed tomography (CT) and magnetic resonance imaging (MRI), ultrasound stands out with its noninvasiveness, affordability, portability, and radiation-free nature (Jiang et al., 2023a; Yang et al., 2021). However, unlike fixed apparatuses such as CT and MRI devices that generate images automatically, the acquisition of standardized and high-quality US images relies on skilled and experienced physicians during traditional free-hand examinations (Sharma et al., 2021; Drukker et al., 2020; Jiang et al., 2020). Therefore, ensuring reproducibility of ultrasound imaging is crucial for enhancing its overall clinical value.

However, the reproducibility of free-hand ultrasound images is influenced by various factors, including operator subjectivity, anatomical sites, acquisition parameter settings and the type of equipment used (Jiang et al., 2022a; Swan et al., 2017; Yoon et al., 2011; Li et al., 2022). During scanning, Ferraioli et al. evaluated the reproducibility of spleen stiffness (SS) and liver stiffness (LS) measurements at various sites using point shear wave elastography (pSWE). They found that the reproducibility of SS measurements depends on operator expertise, and measurements varied significantly across different sites of the same organ (Ferraioli et al., 2014). Similarly, studies on abdominal aortic measurements, Doppler ultrasound measurements, thyroid shear wave elastography (SWE) measurements, and cervical length and width measurements during pregnancy with ultrasound have all revealed poor intra- and/or inter-observer reproducibility (Swan et al., 2017; Matthews et al., 2021; Mikkonen et al., 1996; Valentin and Bergelin, 2002). Karlas et al. reported that the diagnostic accuracy of pSWE is influenced by the angle of the region of interest (ROI), with the lowest variation observed at a perpendicular ROI position centered on the transducer surface (Karlas et al., 2011). Benediktsdottir et al. tested the reproducibility of head-perineum distance (HPD) measurements using two different ultrasound devices and found significant differences between the devices (Benediktsdottir et al., 2018). Similarly, Ellis et al. investigated the instrument bias of aortic diameter measurements obtained with three types of ultrasound scanners (Ellis et al., 1991). Their study, conducted on ten patients with small infrarenal abdominal aortic aneurysms, calculated the limits of agreement between machines.

Due to the low reproducibility of ultrasound image acquisition and measurement, artificial intelligence (AI) tools have been employed recently. Salte et al. developed an AI method based on deep learning to provide fully automated measurements of left ventricular global longitudinal strain, which reduced test-retest variability and eliminated bias between readers in test-retest datasets (Salte et al., 2023). Similarly, Karužas et al. examined the reproducibility of an AI-based automated aortic measurement software, finding it feasible and closely aligned with manual measurements by experts, with improved reproducibility (Karužas et al., 2022). In terms of diagnosis, AI has been widely used to assist physicians to make diagnoses with medical images (Li et al., 2024). However, these methods still rely on clinicians to manually acquire the relevant imaging planes, which introduces both inter-operator and intra-operator variability. In conclusion, there is a growing emphasis on improving the reproducibility and standardization of ultrasound features.

The Robotic Ultrasound Scanning System (RUSS) has gained remarkable traction and interest over the past two decades, transforming the way traditional ultrasound examinations are conducted (Jiang et al., 2023a; Yang et al., 2021; Jiang et al., 2022b; Huang et al., 2023a; Huang et al., 2023b; Zhou et al., 2024). In comparison to traditional free-hand ultrasound procedures, RUSS stands out with its enhanced precision and reproducibility, showing immense potential to reduce intra-operator and inter-operator discrepancies (Gilbertson and Anthony, 2015a; Kojcev et al., 2017). Prior research has investigated the influence of various factors on the reproducibility of RUSS scanning, including force control and ultrasound visual servoing. In the field of RUSS force control, Yang et al. (2021), Huang et al. (2019), Huang et al. (2018) used two force sensors attached to the front face of probe for controlling the contact force and position of the probe. Their results demonstrated that this system achieved a measurement error of less than 1% for volume estimation. For visual servoing, several methods have been developed to enhance robotic perception and autonomy, including feature-based approaches, hybrid approaches, and machine-learning-based approaches (Fei et al., 2023). In the domain of RUSS visual servoing, Zielke et al. combined the segmentation model with an automated robotic ultrasound scanning system, achieving a reduction in thyroid measurement error from 20.85% to 8.23% compared to ultrasound physicians (Zielke et al., 2022). Their study added another layer of evidence supporting the potential of RUSS in transforming ultrasound imaging. However, these studies only focused on contact force or the effects of the image acquisition process.

Recently, Ning et al. proposed a learned-active compliance control strategy based on inverse reinforcement learning to perform simultaneous posture and force control for autonomous RUSS in unstructured environments (Ning et al., 2024). The results showed that the methods improved the stability of different phantoms. However, this approach primarily focuses on force control and lacks sufficient quantitative analysis of the acquired ultrasound images. Dall'Alba et al. introduced an imitation learning method based on Kernelized Movement Primitives by training an autonomous robotic controller using sonographer demonstrations (Dall’Alba et al., 2024). While this approach achieved reproducible force control and ultrasound image quality, it still required human intervention to manually select the upper-level plan, limiting its ability to fully automate the procedure. Deng et al. introduced a multimodal reinforcement learning algorithm with a similarity network to guide automatic scanning, considering factors like force, position, and image quality (Deng et al., 2024). Hsowever, this study lacks a comparative analysis of force control and image quality, and requires physician guidance, large datasets for training, and significant computational resources. It is also limited to virtual environments and phantom models, with no real-world clinical validation. Ning et al. proposed a decoupled control strategy for autonomous vascular ultrasound imaging, utilizing image-guided orientation control and force-guided posture control (Ning et al., 2023). The system demonstrated the ability to autonomously image vessels on arms in various conditions and achieved reproducible imaging. However, this study lacks a comprehensive comparison between RUSS and traditional ultrasound physicians. Furthermore, it does not account for post-processing image analysis.

With the rapid advancement of AI, the synergy between RUSS and post-processing image analysis has emerged as a promising pathway in decision-making within ultrasound imaging (Jiang et al., 2023a; Li et al., 2021; Burke et al., 2020). Current research lacks studies on the integration of such systems. By integrating RUSS with AI technologies, we expect to significantly enhance image analysis reproducibility and observer consistency in ultrasound imaging. Additionally, existing studies have not yet comprehensively compared RUSS with traditional ultrasound physicians, which is one of the key methods for assessing the clinical applicability and value of the system.

In this study, we aim to address the challenges associated with assessing reproducibility in traditional free-hand ultrasound examinations. To achieve this, we have developed a pipeline that integrates a fully autonomous RUSS (auto-RUSS) with a robust ultrasound image post-processing system. Our study focuses on conducting a novel and systematic analysis, comparing the reproducibility and inter-observer agreement of the proposed pipeline with those of assessments made by eight ultrasound physicians with varying levels of expertise. This analysis focuses on crucial aspects including force control, image acquisition, and post-processing image analysis, which encompass image measurement and the utilization of AI-assisted classification.

## 2 Materials and methods

### 2.1 Autonomous robotic ultrasound scanning system (auto-RUSS) design

#### 2.1.1 System setup

**FIGURE 1:**
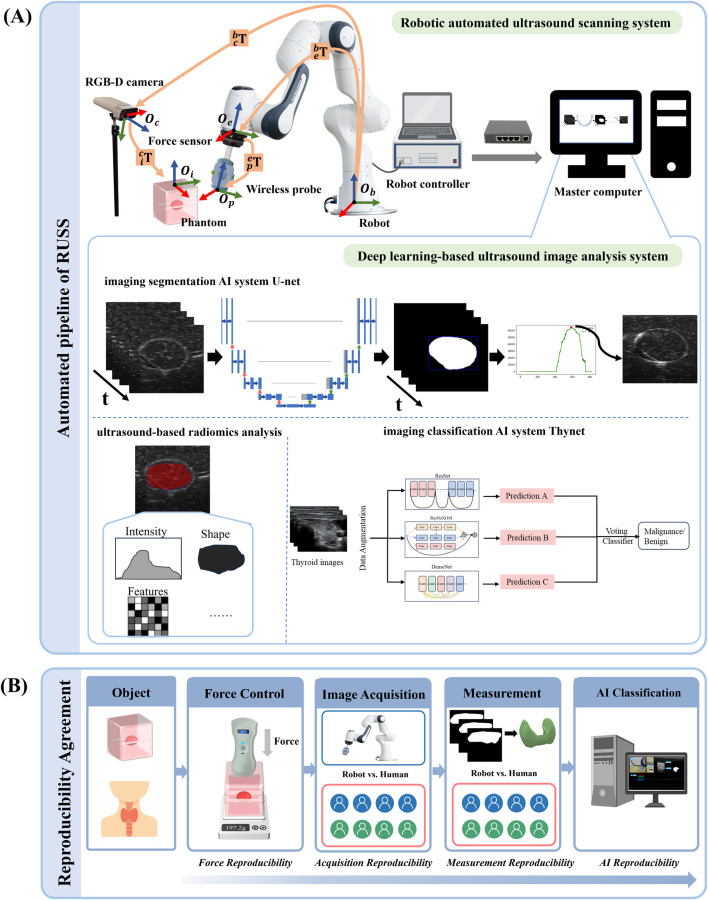
The overall pipeline and the reproducibility and observer consistency. **(A)** The pipeline of the robotic autonomous ultrasound scanning system. The diagram includes the mechanism of the robotic arm, the force controller of the auto-RUSS system, and the deep learning-based ultrasound image analysis system of auto-RUSS. This analysis system consists of a deep learning-based image segmentation AI system (U-net), an ultrasound-based radiomics analysis software, and a classification AI system (Thynet). The red, blue, and green coordinate arrows represent the X-axis, Y-axis, and Z-axis, respectively. The orange arrow indicates the transformation chain of matrices between coordinate systems. **(B)** Workflow for the reproducibility and observer consistency, encompassing intra- and inter-reproducibility of scanning contact force, reproducibility of ultrasound visual servoing image acquisition, reproducibility of image measurement, and reproducibility of AI-assisted tumor classification.

As depicted in Figure 1A, the key components of the system are as follows: a 7-degree-of-freedom robotic arm (Panda, Franka Emika, Germany); a 6-axis force torque sensor (FT 300-S, Robotiq, Canada); a wireless US probe (D5CL, SonoHealth Medical Technologies, China); an RGB-D camera (Azure Kinect, Microsoft, United States); an image and video store system (MaiYing, Guangzhou, China), a tumor phantom (Ningbo Lancet Medical Technology, China) and a thyroid phantom (Model 074, CIRS, United States). The robotic arm is controlled by a Robotic Operating system (ROS) framework. No institutional review board approval was required because only phantoms were used.

#### 2.1.2 Coordinate system transformation for ultrasound robot

Establishing a fully autonomous robotic system entails configuring it to navigate the probe towards the object region along a predetermined trajectory. This is achieved by first aligning the coordinate system of the probe with that of the phantoms.

As depicted in Figure 1A, the robot arm base coordinate system is $O_{b}$ ; the robot arm end flange coordinate system is $O_{e}$ ; the probe coordinate system is $O_{p}$ ; the RGB-D camera coordinate system is $O_{c}$ ; and the coordinate system at the surface target point is $O_{i}$ . Their transformation relationships can be calculated as Equations 1, 2:

Tib=TcbTic (1)

Tpb=TebTpe (2)

where T∈R4×4kj represents the transformation matrix utilized for transferring position from frame k to frame j. The Tib refers to the position and orientation of the phantom in the coordinate system $O_{b}$ of the Franka robotic arm. Similarly, Tcb denotes the transformation relationship between the RGB-D camera $O_{c}$ and the coordinate system $O_{b}$ of the robotic arm. Since the RGB-D camera is fixed in the scene, Tcb can be obtained through the hand-eye calibration method. At any given time, the position and orientation of the phantom in the RGB-D camera’s coordinate system $O_{c}$ can be computed through normal evaluation of point cloud data. The Teb represents the kinematic model of the robotic arm, which can be directly obtained from the Franka Control Interface (FCI) provided by the manufacturer. In addition, Tpe depends on the custom configuration. In this work, the rotation part of Tpe is set to an identity matrix I^3 × 3^, and the translational part is obtained from the custom-designed 3D-printer holder model or can be calculated through calibration with an ultrasound probe. During the process of automatic ultrasound scanning, the detection and position calculation of the phantom target point are completed on the host computer, and then the pose of probe Tpb is aligned with the phantom surface target point Tib .

Once the COS are synchronized, the robotic arm is programmed to navigate the probe to the target point following the planned trajectory. During the scanning process, real-time auto-regulation of the robotic arm’s scanning trajectory is implemented, based on the contact force (force control) and the content of the ultrasound image (ultrasound visual servoing).

#### 2.1.3 Constant contact force based on the force feedback control algorithm

Maintaining constant contact force between the ultrasound probe and surface tissue is vital for high-quality ultrasound imaging (Jiang et al., 2023b). However, varying tissue deformations caused by pressure can lead to changes in the contact force and ultimately affect image quality. To overcome this challenge, we employ a contact force feedback control algorithm to maintain the contact force between the ultrasound probe and the surface tissue at a constant level, which was empirically set to be a constant value of 2.0 N. The robot end effector is positioned vertically to the workbench surface, and after the scan is initiated, the controller moves the robot arm end effector to the object point above the phantom. Once the probe reaches the phantom surface, the contact force is sensed in real time by the force sensor connected between the robot arm and the probe holder, and the force feedback control algorithm is activated.

The force control algorithm is implemented through a Proportional-Derivative (PD) controller on the Z-axis (Table 1; Figure 1A). The controller calculates the force error $F_{error}$ and the change in force error $∆F_{error}$ between the end ultrasound probe contact force $F_{e}$ and the target contact force $F_{goal}$ , and controls the end effector to move on the Z-axis in $Z_{step}$ until the force error is less than the set threshold $F_{thre}$ , thereby maintaining a constant pressure at the end of the robot arm.

**TABLE 1:** Force feedback control algorithm.

Algorithm 1: Force feedback control algorithm
input: $F_{goal}$ , $F_{thre}$ , $K_{p}$ , $K_{d}$ , scale
1. initialization: $F_{error\_d}\leftarrow 0$ ;
2. $F_{e}=getCurrentForce\left(\;\right)$ ;
3. $F_{error}=\left|F_{e}-F_{goal}\right|$ ;
4. while $F_{error}>F_{thre}$ do:
5. if $F_{goal}-F_{e}>0$ then
6. $F_{error}=F_{goal}-F_{e}$ ;
7. $∆F_{error}=F_{error}-F_{error\_d}$ ;
8. $Z_{step}=-K_{p}*F_{error}-K_{d}*∆F_{error}$ ;
9. $F_{error\_d}=F_{error}$ ;
10. else
11. $F_{error}=F_{e}-F_{goal}$ ;
12. $∆F_{error}=F_{error}-F_{error\_d}$ ;
13. $Z_{step}=K_{p}*F_{error}+K_{d}*∆F_{error}$ ;
14. $F_{error\_d}=F_{error}$ ;
15. end if
16. moveEffectorZaxis( $Z_{step}*scale$ );
17. end while

The impedance controller regulates the interaction forces between the robotic end-effector and the patient’s skin during scanning. This controller adjusts the system’s response according to the desired stiffness, damping, and mass properties, thereby ensuring a compliant interaction while maintaining stability. Therefore, we compare the performance of the PD controller with that of the impedance controller.

#### 2.1.4 Deep learning-based ultrasonic image servo and analysis system

As illustrated in Figure 1A, to improve ultrasound image quality, a control algorithm founded on ultrasound visual servoing was formulated. This technique involves utilizing visual feedback from ultrasound images to guide probe motion, enabling real-time adjustments to the scanning path and ensuring the target area remains centered, leading to optimal image acquisition.

Specifically, using a pre-trained ultrasound segmentation model U-Net, we extract real-time binary labels for the target area, which are composed of target labels (1) and background (0). When the target is located on either side of the image, we calculate the error ∆d between the center of the target and the center of the image. Then, the controller moves the end effector along the y-axis on the y-z plane until the error ∆d is less than the threshold value $∆d_{thre}$ (Table 2).

**TABLE 2:** Ultrasonic image servo algorithm.

Algorithm 2: Ultrasonic image servo algorithm
input: $∆d_{thre}$
1. $∆d={object}_{x}-{center}_{x}$ ;
2. while $\left|∆d\right|>∆d_{thre}$ do:
3. if ∆d>0 then
4. moveEffectorYaxis( ∆d*K );
5. else
6. moveEffectorYaxis( −∆d*K );
7. end if
8. $∆d={object}_{x}-{center}_{x}$ ;
9. end while

Upon contact of the probe with the phantom, data acquisition is initiated, with both the raw ultrasound image video stream and the real-time analysis results from U-Net being recorded and stored. After the scan is completed, the maximum cross-sectional area of the target, as determined by the binary mask, is automatically selected, and the corresponding optimal raw ultrasound image is obtained. Finally, the optimal frame is input into the developed feature extraction system for further evaluation.

### 2.2 Data collection and image post-processing

Two phantoms were employed for data collection, one is a tumor phantom, and another is a thyroid phantom simulated human thyroid with a benign nodule located. The tumor phantom (Ningbo Lancet Medical Technology, China) used in this study is designed to simulate a lesion adjacent to blood vessels. The phantom, measuring 15 cm × 12 cm × 5 cm, housed a centrally located simulated tumor with a diameter of 1 cm, and incorporated two simulated vasculature structures aligned along the longitudinal dimension. Its purpose is to evaluate the impact of auto-RUSS and ultrasound physicians with varying levels of experience on the reproducibility of radiofrequency (RF) measurements under identical ultrasound acquisition parameters. And the thyroid phantom (Model 074, CIRS, United States) used in this study contains a slightly enlarged thyroid gland positioned within an anthropomorphic neck. The chin and clavicle are provided as external anatomical landmarks. The phantom provides the trachea, internal jugular vein and common carotid artery as internal anatomical landmarks. Each thyroid lobe contains one cyst and one isoechoic stiff lesion. Various nodules can be manufactured within the thyroid gland on a custom basis. All materials are formulated to be ultrasonically realistic.

As depicted in Figure 1A, a master computer facilitated the execution of the data acquisition and deep learning-based image post-processing workflow. This pipeline incorporated the utilization of an ultrasound-based radiomics analysis software (Ultrasomics-Platform, version 2.1, Guangzhou, China) (Li et al., 2022), a deep learning-based imaging segmentation AI system U-Net and classification AI system ThyNet (Peng et al., 2021). ThyNet is composed of three renowned backbone networks running in parallel: ResNet, DenseNet, and ResNeXt. ResNet is a celebrated backbone network in the field of computer vision, with the proposed skip connections and network paradigm still widely used in various models today. DenseNet employs dense connections to enhance the associativity between different stages of the network. ResNeXt uses group convolutions to better enhance the semantic information of features. By jointly considering the output features of the three backbone networks, more accurate image features can be captured. The specifications of the three networks are ResNet101, DenseNet201, and ResNeXt101, respectively.

### 2.3 Reproducibility and observer consistency: auto-RUSS pipeline vs. traditional ultrasound physicians

After establishing the Auto-RUSS system, its reproducibility was compared with those of traditional ultrasound physicians. Eight physicians were divided into two groups based on their experience levels: the expert group (expert 1–4) and the non-expert group (non-expert 1–4). The expert group comprised physicians with more than 5 years of experience in thyroid ultrasound, who had completed both the standardized national residency training and specialized ultrasound training. These experts review thyroid ultrasound images from approximately 800 patients annually. In contrast, the non-expert group consisted of radiologists with less than 3 years of experience in performing ultrasound scans. These radiologists had undergone a 3-year standardized national residency training program, which included comprehensive instruction on thyroid ultrasound examinations, and they evaluate thyroid ultrasound images from approximately 600 patients per year. The study compared the reproducibility of contact force, image acquisition, image measurement, and AI-assisted classification between the Auto-RUSS system and the physician groups.

#### 2.3.1 Reproducibility of scanning contact force

We first conducted experiments on a tissue-mimicking phantom. To assess intra-operator reproducibility of the scanning contact force, we initially compared the performance of the PD controller and the impedance controller. The auto-RUSS was tested with both controllers at six different scanning speeds (1.0 mm/s, 3.0 mm/s, 5.0 mm/s, 7.0 mm/s, 9.0 mm/s and 11.0 mm/s). Each speed was tested ten times, with the system maintaining a consistent contact force of 2.0 N throughout. In addition, test-retest reproducibility was evaluated through ten unique scans along the longitudinal dimension of the phantom at a speed of 3.0 mm/s. For evaluating inter-operator reproducibility, as depicted in Figure 1B, both the auto-RUSS and eight ultrasound physicians (four experts and four non-experts) were asked to independently scan along the longitudinal direction of the phantom ten times.

To further validate the feasibility of the proposed system, we extended the experiment to a human thyroid. Both the auto-RUSS and the eight ultrasound physicians were asked to independently perform ten scans along the longitudinal direction of the thyroid of a volunteer. During each scan, the contact force and its variation were continuously monitored using a force sensor (FT 300-S, Robotiq, Canada).

#### 2.3.2 Reproducibility of visual servoing acquisition

To assess the reproducibility of the ultrasound visual servoing image acquisition, both the auto-RUSS and the physicians independently captured the maximum cross-section of the centrally positioned simulated tumor in the phantom ten times. For objective analysis, we adopted quantitative radiomics features, as suggested by prior research (Li et al., 2022), to facilitate reproducibility analysis. Employing the Ultrasomics-Platform (version 2.1, Guangzhou, China) (Figure 1A), we extracted 5,408 ultrasound features for each image. A total of 4,614 non-zero features were identified from all US images and selected for subsequent comparisons, including original features (n = 111, 2.41%), co-occurrence of local anisotropic gradient orientation (CoLIAGe) features (n = 570, 12.35%), wavelet features (n = 302, 6.55%), Shearlet features (n = 2,944, 63.81%), Gabor features (n = 547, 11.86%), and Pyramid Local Binary Pattern (PLBP) features (n = 140, 3.03%).

This analysis included a total of 90 complete original ultrasound images. The coefficient of variation (COV) was utilized as a metric to evaluate the reproducibility of the acquired features.

#### 2.3.3 Reproducibility of image measurement

For the auto-RUSS, measuring a certain target’s volume involve segmentation and 3D reconstruction techniques, both are essential aspects of autonomous image analysis. In order to compare the reproducibility of measurements on the same target in clinical settings between the auto-RUSS and physicians, a simulated human neck and thyroid phantom was employed. Both the auto-RUSS and physicians performed ten scans on the phantom, with relevant data to calculate the thyroid volume meticulously recorded. The auto-RUSS utilized a deep learning segmentation U-Net model (Figure 1A), continuously segmenting the thyroid during the scanning process and recording segmented images. The 3D reconstruction rendering and volume calculation of the thyroid were accomplished using the 3D Slicer software (version 5.6.1). In contrast, physicians were instructed to individually measure the height (L), width (W), and thickness (D) of the left and right thyroid lobes following traditional procedures and calculate the thyroid volume according to the following formula (Liang et al., 2017):

$$V\left(\text{ml}\right)=0.479\times D\times W\times L\left(\text{mm}\right)/1000.$$

The volume of the thyroid reconstructed from CT scans is regarded as the gold standard.

#### 2.3.4 Reproducibility of classification

Classification is a vital task in medical decision-making, offering direct assistance in tasks such as benign versus malignant diagnosis and survival prognosis. To assess classification reproducibility, a thyroid phantom was employed. The auto-RUSS and eight physicians respectively conducted ten scanning to capture the maximum cross-sectional area of the nodule. Subsequently, the images were input into ThyNet model (Peng et al., 2021) (Figure 1A), which assigns classification and malignant probability values to the nodule.

### 2.4 Statistical analysis

To assess the reproducibility comparison between auto-RUSS pipeline and ultrasound physicians, the participants were partitioned into three groups: the auto-RUSS group; the non-expert group, comprising four physicians with 0–4 years of experience; and the expert group, made up of four physicians with over 4 years of experience. All data were expressed as mean ± SD. For the reproducibility of probe contact force, a boxplot was employed to depict the median, interquartile range, maximum and minimum. For the reproducibility of ultrasound visual servoing, which was quantified by the extracted radiomics features, the coefficient of variation (COV) was computed for each radiomics feature according to the following formula:

$$COV=\frac{SD}{Mean}\times 100\%$$

SD is the standard deviation of the feature values. If Mean is zero, the statistics of this radiomics feature are removed.

Based on the COV, the reproducibility of ultrasound radiomics features was classified into three levels: good (COV ≤10%), moderate (10% < COV ≤20%), and poor (COV >20%) (Li et al., 2022). For the reproducibility of measurement, a boxplot and COV were employed. Wilcoxon signed-rank test was used to investigate whether there is a significant difference between the measured thyroid volume values and the gold standard. For the reproducibility of AI-assisted tumor classification, a boxplot and COV were used to evaluate the model output probability values. All statistics were performed using Python (version 3.8).

## 3 Results

### 3.1 Autonomous pipeline of RUSS

**FIGURE 2:**
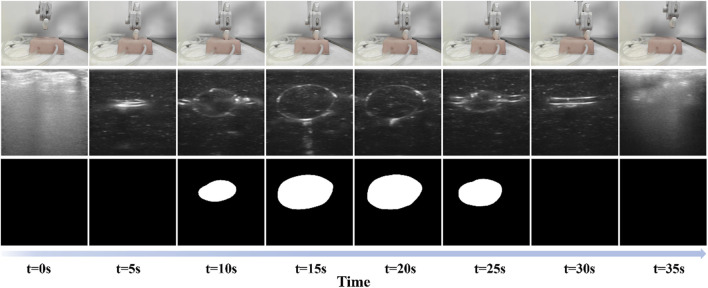
Scanning process of the proposed auto-RUSS pipeline. The first row represents the real-world scene, the second row shows the acquired real-time ultrasound images, and the third row displays the autonomously segmented ROI. Each column corresponds to a different time point.

To evaluate the methods and algorithms proposed in this study, experiments were conducted using the proposed auto-RUSS depicted in Figure 1. The master computer obtained real-time point cloud data with a frequency of 30 Hz using an RGB-D camera. The YOLOv3 detection model was employed to update the phantom’s pose, enabling the planning of the scanning trajectory. The master computer communicated with the Franka robotic arm’s controller at a frequency of 1 kHz, ensuring stable motion and feedback on robot poses and end-effector contact force. The probe was then adjusted to the target point through coordinate system transformation, and the scan was carried out along the planned trajectory, conducting image post-processing (Figure 2). During the scan, the robotic arm’s trajectory was regulated in real-time based on contact force and ultrasound image content, ensuring the reproducibility of both contact force and image quality through image post-processing.

The ultrasound settings used in this study were as follows: the frequency was set to 7.5 MHz, the gain was adjusted to 105 dB, the focus was set at 2 cm, the dynamic range (DR) was set to 80, the mechanical index (MI) was 0.7.

### 3.2 Reproducibility of probe contact force

**FIGURE 3:**
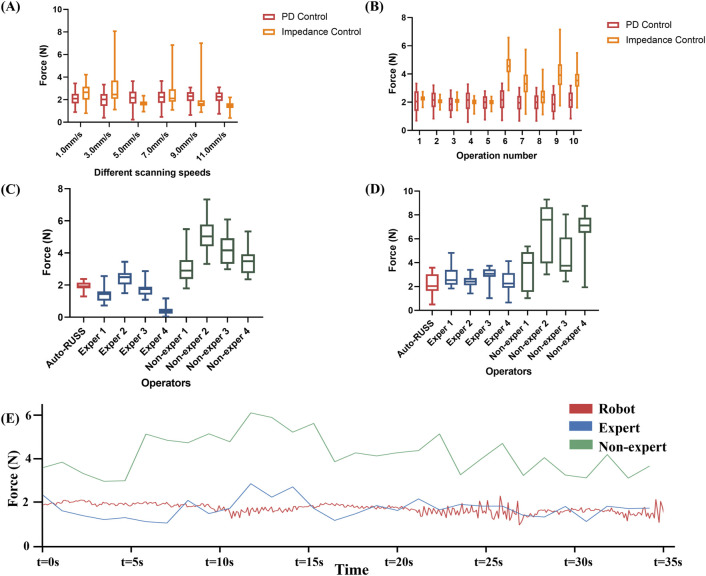
The reproducibility of contact force was examined through the following experiments: **(A)** the contact force during repetitive scans at varying speeds by the robotic ultrasound system with PD controller and impedance controller, **(B)** the contact force during repetitive scans by the robotic ultrasound system with PD controller and impedance controller, **(C)** a comparison experiment on a phantom of the contact force between the auto-RUSS group, expert groups, and non-expert groups, **(D)** a comparison on a volunteer of the contact force between the auto-RUSS group, expert groups, and non-expert groups, and **(E)** Force measured at 3 mm/s scanning speeds. The expected contact force is 2.0 N.

For evaluating intra-operator reproducibility of the scanning contact force in the auto-RUSS group, as shown in Figure 3A, both the force feedback control algorithms kept the end-probe contact force near the desired value (2 N) at different speeds to simulate different clinical scan conditions. However, while the impedance control exhibited smoother overall behavior at some speed values, it also presented more extreme values, particularly at scanning speeds of 3.0 mm/s, 7.0 mm/s, and 9.0 mm/s. For the PD control, the contact forces for scanning at 1 mm/s, 3 mm/s, 5 mm/s, 7 mm/s, 9 mm/s, and 11 mm/s were 2.2 ± 0.5 N, 2.1 ± 0.6 N, 2.2 ± 0.6 N, 2.2 ± 0.6 N, 2.1 ± 0.5 N, and 2.1 ± 0.5 N, respectively. This indicated that the PD control algorithm could effectively regulate the contact force of the probe. In addition, considering safety and comfort, the phantom was scanned ten times at a speed of 3 mm/s to evaluate the test-retest reproducibility. As illustrated in Figure 3B, for the PD control, the auto-RUSS system maintained the contact force predominantly within the range of 1.0–3.0 N, demonstrating good intra-operator reproducibility, for the impedance control. In contrast, while impedance control appeared more stable during the first five scans, it became increasingly unstable in subsequent tests. Therefore, we selected the PD control for further experimental analysis.

For inter-operator reproducibility, we first conducted experiments on a tissue-mimicking phantom. The force control capabilities of the auto-RUSS group, expert group, and non-expert group were compared and analyzed. The COV of the contact force in the auto-RUSS group was lowest with mean of 1.9 N. Subsequent experiments on the human thyroid exhibited a similar trend, with the COV of contact force in the auto-RUSS group being the lowest, with a mean of 2.1 N. These results indicated that the auto-RUSS group maintained the contact force at an optimal level with high reproducibility (Figures 3C, D; Table 3).

**TABLE 3:** The comparison of the contact force between the auto-RUSS group, expert groups, and non-expert groups.

	Phantom experiment			Human experiment		
Groups	Mean (/N)	SD	COV	Mean (/N)	SD	COV
Auto-RUSS	1.9	0.2	0.11	2.1	0.5	0.09
Expert 1	1.4	0.5	0.36	2.8	0.8	0.29
Expert 2	2.4	0.5	0.21	2.4	0.5	0.20
Expert 3	1.7	0.4	0.24	3.0	0.6	0.21
Expert 4	0.4	0.2	0.50	2.4	1.0	0.41
Non-expert 1	3.1	0.8	0.26	3.4	1.6	0.48
Non-expert 2	5.1	1.0	0.20	6.6	2.4	0.36
Non-expert 3	4.2	0.9	0.21	4.6	1.8	0.39
Non-expert 4	3.4	0.8	0.24	7.0	1.3	0.19

Finally, we visualized the changes in contact force during a scan across different groups. In the robot group, the parameters were set to a contact force of 2 N and a speed of 3 mm/s. The variations in force in the robot group were significantly more stable compared to the expert and non-expert groups (Figure 3E).

### 3.3 Reproducibility of ultrasound visual servoing image acquisition

**FIGURE 4:**
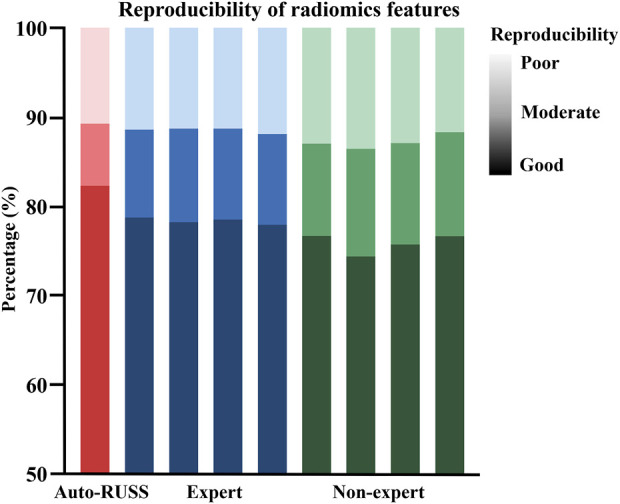
The reproducibility comparison of image acquisition, which was quantified by the extracted radiomics features, between the auto-RUSS group using the ultrasound visual servoing image acquisition method and traditional ultrasound imaging conducted by physicians of various experience levels. Based on the COV, the reproducibility of ultrasound radiomics features was classified into three levels: good (COV ≤10%), moderate (10% < COV ≤20%), and poor (COV >20%), and the deeper the color, the higher the reproducibility.

Figure 4 depicted the comparison of reproducibility between the auto-RUSS group, which utilized ultrasound visual servoing for image acquisition, and the physician groups, who obtained ultrasound images using traditional methods. The analysis of the radiomics features of the images indicated that the auto-RUSS group achieved a superior level of reproducibility, with 75.73% of radiomics features demonstrating good reproducibility. This outcome was marginally better than that of the expert groups, which had a mean of 73.43% (with a range of 72.52%–75.08%). Both the auto-RUSS group and the expert groups had higher levels of good reproducibility than the non-expert groups (mean 70.70% with a range of 69.40%–71.69%).

### 3.4 Reproducibility of measurement

**FIGURE 5:**
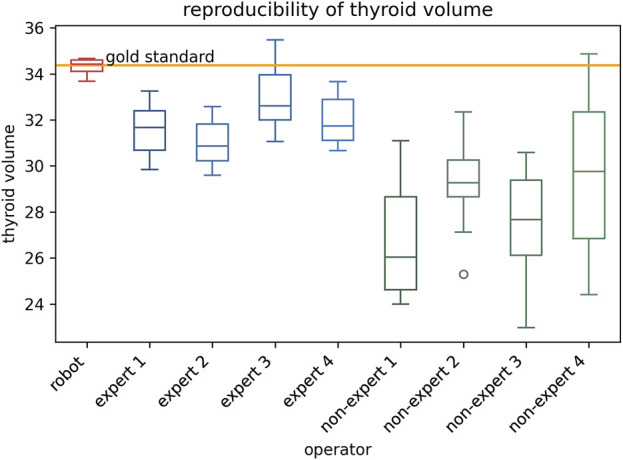
The reproducibility comparison between the auto-RUSS group using deep learning method and physicians of various experience levels in thyroid volume measurement based on ultrasound image.

The results of thyroid volume measurement reproducibility are presented in Figures 5, 7C and Table 4. The auto-RUSS group demonstrated the highest reproducibility with a coefficient of variation (COV) of 0.01, while the measurements from the non-expert groups exhibited the greatest instability, with the highest SD and COV values. There was no statistically significant difference between the thyroid volume measured by the auto-RUSS group and the gold standard (34.3 mL vs. 34.5 mL, P = 0.285). This indicated that the auto-RUSS could accurately measure the anatomical structure with high repeatability, thereby demonstrating a high level of reliability. The expert groups exhibited superior reproducibility compared to the non-expert groups (COV 0.03-0.05 vs. 0.07-0.13), but both were significantly less accurate than the gold standard (31.0–33.0 mL vs. 26.8–29.7 mL vs. 34.5 mL, P < 0.05).

**TABLE 4:** The comparison of the thyroid volume measurement between the auto-RUSS group, expert groups, and non-expert groups.

	Mean (/mL)	SD	COV	P-value
Auto-RUSS	34.3	0.3	0.01	0.285
Expert 1	31.6	1.1	0.04	0.005^*^
Expert 2	31.0	1.1	0.03	0.005^*^
Expert 3	33.0	1.5	0.05	0.022*
Expert 4	32.0	1.1	0.03	0.005^*^
Non-expert 1	26.8	2.7	0.10	0.005^*^
Non-expert 2	29.2	1.9	0.07	0.005^*^
Non-expert 3	27.5	2.3	0.09	0.005^*^
Non-expert 4	29.7	3.8	0.13	0.007^*^

P-values were calculated by comparing the measurements with the thyroid volume reconstructed after CT (34.48 mL).

*P < 0.05.

### 3.5 Reproducibility of classification

**FIGURE 6:**
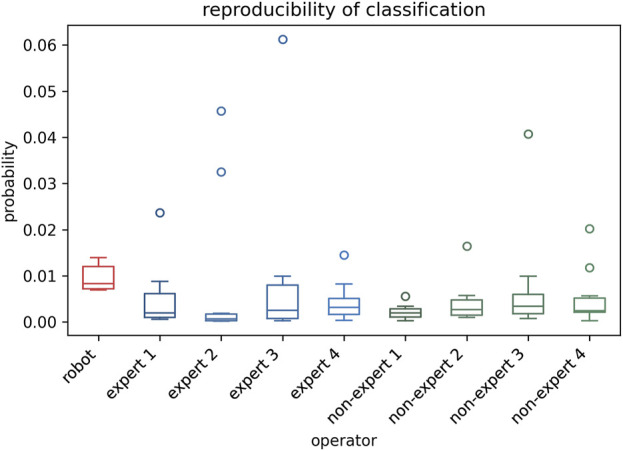
The reproducibility of the auto-RUSS, expert, and non-expert groups in AI-assisted tumor classification. The ordinate represents the malignant probability values outputted by the model.

**FIGURE 7:**
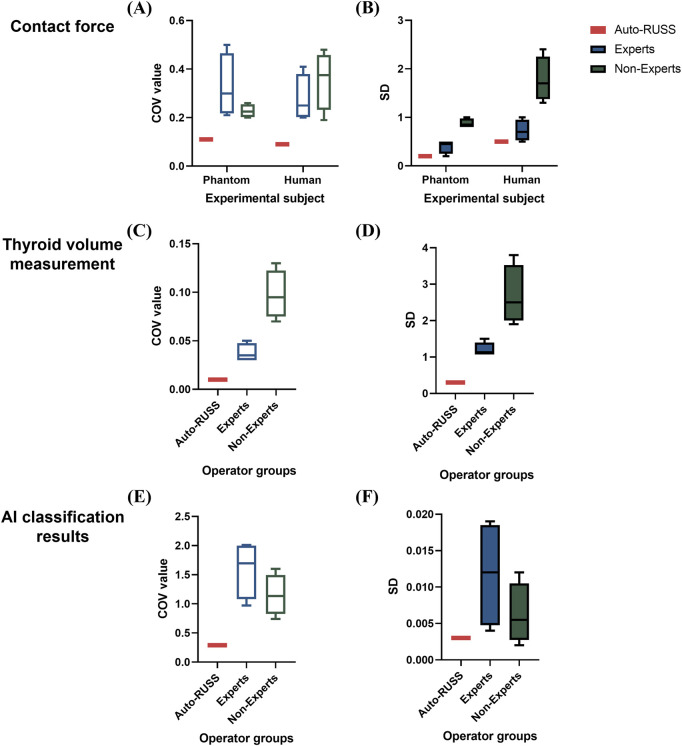
Boxplots of the coefficient of variation (COV) and standard deviation (SD) values across different experiments comparing the auto-RUSS group, expert group, and non-expert group. **(A, B)** Boxplots of COV and SD values for the reproducibility of contact force measurements on both the phantom and human subjects. **(C, D)** Boxplots of COV and SD values for the reproducibility of thyroid volume measurements. **(E, F)** Boxplots of COV and SD values for the reproducibility of AI classification results.

The stability of classification reproducibility was quantified by measuring the COV and SD of the malignant probability values output by the AI model (Figures 6, 7E). For the same benign lesion, all sets of images acquired by three groups were accurately classified. Regarding the predicted probability values, the auto-RUSS group exhibited the lowest COV (0.2874) and SD (0.003) value among all groups (Table 5). Additionally, based on Figure 6, it was evident that all groups, except for the auto-RUSS group, had outliers, providing further evidence of the higher reproducibility in the auto-RUSS group. This demonstrated that images acquired by auto-RUSS and analyzed by AI yield more consistent results, further advancing the integration of ultrasound and AI-based image analysis.

**TABLE 5:** The comparison of the classification results between the auto-RUSS group, expert groups, and non-expert groups.

	True label	Pred	Mean	SD	COV
Auto-RUSS	B	B	0.009	0.003	0.29
Expert 1	B	B	0.005	0.007	1.42
Expert 2	B	B	0.008	0.017	1.97
Expert 3	B	B	0.009	0.019	2.01
Expert 4	B	B	0.004	0.004	0.97
Non-expert 1	B	B	0.002	0.002	0.74
Non-expert 2	B	B	0.004	0.005	1.09
Non-expert 3	B	B	0.007	0.012	1.60
Non-expert 4	B	B	0.005	0.006	1.18

B = benign.

## 4 Discussion

In this research, we developed a robotic ultrasound scanning system aiming to enhance the reproducibility of ultrasound scanning while providing a fully autonomous pipeline for ultrasound image acquisition and analysis. To the best of our knowledge, this is the first study that offers a comprehensive evaluation of a robotic ultrasound system’s reproducibility in comparison to traditional ultrasound practitioners. This examination encompasses four crucial aspects: force control, image acquisition, image measurement, and AI-assisted classification. By minimizing operator subjectivity, the auto-RUSS enhances the reproducibility of ultrasound features, thus establishing a robust basis for the development of highly generalized ultrasound AI models.

Prior research has revealed that varying probe contact force levels can impact the reproducibility of ultrasound images and elastography measurement (Sai et al., 2022; Zhang et al., 2022; Wang et al., 2021; Wang et al., 2019; Tan et al., 2022; Kaminski et al., 2020). Our auto-RUSS was established primary based on force control. The result suggests that the algorithms can maintain the contact force at different scanning speeds. In clinical practice, different contact forces may lead to change in ultrasound image features, thus directly affecting diagnostic outcomes (Tan et al., 2022). It was observed that compared to the other groups, the non-expert group tended to use larger forces for image acquisition and exhibited higher variability, suggesting a significant operator-dependency of contact force. Furthermore, we validated the feasibility of the system on human subjects, and the auto-RUSS group also demonstrated the most stable force control, further confirming the system’s effectiveness.

Image acquisition based on ultrasound visual servoing wields a significant influence on various downstream tasks including segmentation, 3D reconstruction, and classification, all of which are integral components of comprehensive medical analysis and procedures (Zielke et al., 2022; Liu et al., 2023; Bi et al., 2022). The analysis of ultrasound radiomics features showed that the images acquired by the auto-RUSS group had the highest proportion of reproducible features, suggesting that ultrasound visual servoing-based acquisition significantly enhances feature reproducibility.

The reproducibility of ultrasound image measurement was subsequently analyzed. A thyroid phantom was utilized, and its volume was repeatedly measured. During this process, the robot employed automatic segmentation and 3D reconstruction techniques, both crucial in image analysis. The results indicated that the thyroid volume measured by the auto-RUSS was the only that has no statistically significant difference with the gold standard among all groups, and reproducibility was superior to the other groups. This highlights the advantage of the robot arm in image processing and demonstrates its potential in clinical applications, such as measurements of certain anatomical sites.

In further exploring the clinical utility of diagnostic AI models, specifically AI-assisted classification, our findings suggest that images obtained by the auto-RUSS show superior reproducibility compared to images manually acquired. The autonomous pipeline, therefore, presents itself as a transformative technology with the potential to significantly enhance ultrasound image reproducibility and lessen operator dependence. This comprehensive approach not only alleviates the workload of ultrasound physicians but also bolsters the efficiency of the examination process. The auto-RUSS can assist physicians in accurately positioning the ultrasound probe, measuring lesion sizes, and identifying potential abnormalities based on the AI analysis system, enabling more proactive and accurate detection of potential health issues.

Currently, international evaluations of ultrasound robotic systems primarily focus on single metric such as force reproducibility or the quality of acquired ultrasound images. For example, Matthew et al. assessed the stability of robotic systems by measuring the pressure exerted by the end effector (Gilbertson and Anthony, 2015b), while Jiang et al. evaluated ultrasound image quality using confidence maps (Jiang et al., 2020). Additionally, Risto et al. compared the consistency of image acquisition and measurement between human-operated and robotic systems (Kojcev et al., 2017). However, there is still a lack of standardized evaluation criteria, particularly for unified human-machine assessment of critical indicators such as force stability and image quality reproducibility. Our study offers an objective and standardized evaluation method to assess the performance of ultrasound robotic systems on these key metrics.

The Auto-RUSS system not only provides a robust reference framework for future research but also establishes a foundation for the clinical adoption and application of ultrasound robotic systems. By automating the ultrasound scanning process, Auto-RUSS simplifies operations, particularly in resource-limited environments or high-volume clinical settings where manual scanning is labor-intensive and time-consuming. Additionally, ultrasound examinations are physically demanding for operators and often contribute to occupational health issues such as neck, shoulder, and lower back pain. Integrating such a system into clinical practice has the potential to alleviate the physical burden on clinicians. Furthermore, ultrasound diagnosis is inherently subjective, with variability in diagnostic outcomes among physicians. Auto-RUSS has the potential to address this challenge by minimizing operator-dependent variability, thereby enhancing diagnostic consistency and accuracy—both of which are crucial for reliable clinical decision-making. Additionally, by enhancing repeatability and reducing the need for rescans due to suboptimal imaging, Auto-RUSS could improve patient throughput and reduce waiting times. By enabling standardized and reproducible imaging, Auto-RUSS may also lower healthcare costs by reducing reliance on highly skilled operators and mitigating variability-related expenses associated with misdiagnoses or repeated procedures. Future research should comprehensively evaluate the system’s impact on healthcare efficiency and patient care quality to further support its clinical integration.

This study has a few limitations. First, as the volunteer included in this study was healthy individuals, human experiments were only conducted with respect to the reproducibility of force. However, we have verified the feasibility of this system in real-world settings. Future plans involve conducting further experiments on a larger and more diverse group of volunteers, including those with and without thyroid nodules, to validate the reproducibility of the system in subsequent stages. Second, this study was limited to the use of a single ultrasound machine and probe, and the effects of devices from different manufacturers were not compared, which limits the generalizability of our findings across systems from other manufacturers. Previous research has shown that using equipment from the same manufacturer can enhance study reproducibility by minimizing instrument-induced variability in feature measurements (Li et al., 2022). However, the impact of different ultrasound devices on the reproducibility of the robotic arm system remains unexplored. Future studies should investigate the performance of the Auto-RUSS system across a variety of ultrasound devices from different manufacturers to enhance its robustness and ensure its broad applicability in diverse clinical environments.

In conclusion, we have developed a robotic arm ultrasound scanning system (auto-RUSS), based on force feedback control, ultrasound image visual servoing to achieve a fully autonomous pipeline for ultrasound image acquisition and analysis. Through comprehensive human-machine comparison experiments, the system was shown to effectively improve the reproducibility of ultrasound images and minimize human-induced variability. Our system can provide high-quality ultrasound image data for developing and constructing stable AI models, thereby improving their generalizability. In the future, we will develop scanning systems for various organs and validate their effectiveness on real-world volunteers.

## Data Availability

The raw data supporting the conclusions of this article will be made available by the authors, without undue reservation.
